# Factors associated with testicular self-examination among unaffected men from multiple-case testicular cancer families

**DOI:** 10.1186/1897-4287-7-11

**Published:** 2009-05-29

**Authors:** Susan T Vadaparampil, Richard P Moser, Jennifer Loud, June A Peters, Mark H Greene, Larissa Korde

**Affiliations:** 1 Division of Population Sciences, Moffitt Cancer Center, Tampa, Flordia, USA; 2 Division of Cancer Control and Population Sciences, National Cancer Institute, Rockville, Maryland, USA; 3 Clinical Genetics Branch, Division of Cancer Epidemiology and Genetics (DCEG), National Cancer Institute, Rockville, Maryland, USA

## Abstract

**Background:**

The lifetime testicular cancer (TC) risk in the general population is relatively low (~1 in 250), but men with a family history of TC are at 4 to 9 times greater risk than those without. Some health and professional organizations recommend consideration of testicular self-examination (TSE) for certain high-risk groups (e.g. men with a family history of TC). Yet little is known about factors associated with TSE behaviors in this at-risk group.

**Methods:**

We collected information on this subject during an on-going NCI multidisciplinary, etiologically-focused, cross-sectional Familial Testicular Cancer (FTC) study. We present the first report specifically targeting TSE behaviors among first- and second-degree relatives (n = 99) of affected men from families with ≥ 2 TC cases. Demographic, medical, knowledge, health belief, and psychological factors consistent with the Health Belief Model (HBM) were evaluated as variables related to TSE behavior, using chi-square tests of association for categorical variables, and t-tests for continuous variables.

**Results:**

For men in our sample, 46% (n = 46) reported performing TSE regularly and 51% (n = 50) reported not regularly performing TSE. Factors associated (p < .05) with regularly performing TSE in multivariate analysis were physician recommendation and testicular cancer worry. This is the first study to examine TSE in unaffected men from FTC families.

**Conclusion:**

The findings suggest that, even in this high-risk setting, TSE practices are sub-optimal. Our data provide a basis for further exploring psychosocial issues that are specific to men with a family history of TC, and formulating intervention strategies aimed at improving adherence to TSE guidelines.

## Introduction

Although testicular cancer (TC) accounts for only 1% of all male cancers, it is the most common cancer in younger men aged 20–35 years old, with about 8,090 new cases estimated to occur during 2008 in the U.S. [1]. TC incidence rates have been rising since the mid-twentieth century, especially among white males [2], while the rates of many other cancers have been decreasing. Although 5-year survival for TC has increased over the past several decades, long term survival remains related to stage at diagnosis [3], with localized TC 5-year survival rates of 99.5%, *versus *70.1% for metastatic TC [4]. In addition, earlier stage disease often requires less extensive treatment. Thus, early detection may minimize both TC-associated morbidity and mortality [5-7].

Leading health organizations debate the efficacy of testicular self-examination (TSE) in reducing TC related mortality [8], particularly with the advent of chemotherapeutic regimens that are highly-effective even for men with advanced disease during the last several decades. However, this decrease in mortality is accompanied by an increase in treatment-related morbidity, particularly for those cancers that are diagnosed at later stages [6,9]. A recent review found little shift of the approximate 5-month delay from onset of TC symptoms to surgical diagnosis over the past seventy-five years [10]. While TSE may not markedly improve the already high survival rates related to TC, early diagnosis does bring significant potential to reduce treatment-related morbidity, since surgery plus close surveillance is a viable management option for many early-stage patients [10]. Education and instruction for men on the normal shape and texture of the testicles, plus information regarding signs and symptoms associated with TC could be a critical component in reducing treatment delay [11,12].

Additionally, as epidemiological research identifies specific TC risk factors, some organizations have refined their screening recommendations to focus on high-risk groups. Established TC risk factors include cryptorchidism (undescended testes), prior contralateral TC and testicular intra-epithelial neoplasia. Testicular cancer risk is also increased in certain genetic conditions, such as Klinefelter and androgen insensitivity syndromes. Other conditions that may increase TC risk include urogenital malformations, testicular dysgenesis, testicular atrophy, history of infertility, and, possibly, exposures to a variety of etiologic agents during key developmental periods [13]. Perhaps most importantly, family history is known to be an important independent risk factor. Approximately 1–3% of men with testicular cancer report the presence of another affected male in their family. Sons of men with TC have a four- to six-fold increase in risk of TC, and brothers carry an eight- to ten-fold increase in the risk when compared with the general population [14-16].

The American Cancer Society recommends that men with known risk factors such as family history seriously consider performing TSE regularly. This approach to modifying general population early cancer detection practices for high-risk populations is not unique to men with a familial risk for TC. For example, the National Comprehensive Cancer Network recommends routine breast self-examination (BSE) for women at increased risk of hereditary breast and ovarian cancer (HBOC) [17], despite randomized controlled trial data refuting the efficacy of BSE in reducing breast cancer mortality [18]. In addition to the possibility that TSE recommendations for self-examination may reduce chemotherapy-related morbidity by increasing the proportion of early-stage TC diagnosed in high-risk men, there may also be further benefit in providing at-risk individuals with a management strategy that involves them directly in their own care, and gives them an enhanced sense of control over their lives [10].

While TSE practices among general population young males have been extensively studied [2,19-26], there are no published data specific to TSE practices among multiple-case family members.

We present an analysis of data collected from participants in a National Cancer Institute (NCI) multidisciplinary, etiologically-focused study of familial testicular cancer (FTC). Its primary aims include [1] identifying FTC susceptibility genes; [2] characterizing the FTC syndrome clinical phenotype; and [3] evaluating psychosocial and behavioral issues resulting from being a member of a family at increased risk of testicular germ cell tumor (TGCT). Segregation analysis has suggested that an autosomal recessive genetic model best explains the distribution of disease in FTC families although, to date, no specific TC susceptibility genes have been identified [27]. The current exploratory sub-study describes the TSE practices of unaffected at-risk men from multiple-case families, and characterizes key demographic, medical, knowledge, health belief, and psychological factors associated with TSE using Health Belief Model (HBM) constructs and terminology [28]. Specifically, we included the HBM variables of perceived benefits, barriers, susceptibility and severity. As previously discussed, TSE may improve high-risk men's sense of control over their own health [10,29]. Thus, to examine this association we assessed health locus of control. Finally, previous behavioral research has emphasized the role of affect in health behaviors [30], thus we included the psychological variables of cancer worry and distress.

## Materials and methods

### Study Population

The 99 TSE sub-study participants were members of 47 families enrolled on an NCI Clinical Genetics Branch (CGB) IRB-approved protocol (#02-C-0178) [31]. Families were eligible for the main study if they had ≥ 2 confirmed cases of TC, or a single family member with bilateral TC. Eligible individuals included all TC cases age > 12 years, their first- and second-degree relatives), and blood relatives who either linked the two TC cases or who had themselves been diagnosed with cancer other than TC. In addition, spouses were included if they had children ≥ 12 years old who were study participants. Participants were ascertained through multiple referral mechanisms: 62/99 (63%) from healthcare providers (primarily physicians and genetic counselors), in response to mailed recruitment letters; 15/99 (15%) from the Testicular Cancer Resource Center (TCRC) , a patient advocacy group,; 13/99 (13%) self-referred through our study website ; and 9/99 (10%) from other sources. Unaffected males > age18 who were first- or second-degree relatives of a TC case were included in the current TSE analysis.

### Data Collection

Data were collected *via *a mailed, written questionnaire (the Lifestyle and Attitudes Questionnaire [LAQ]), developed specifically for the FTC study. The present analysis was limited to those LAQ items pertaining to TSE and the relevant independent variables. These independent variables included items and scale scores from standardized, validated psychometric instruments, as well as items developed specifically for this study.

### Measures

For this analysis, we chose variables of theoretical and practical interest related to predicting health behavior, as well as other factors which have been previously-implicated as related to cancer self-examination behavior.

#### Demographic and Medical Characteristics

Various sociodemographic and medical characteristics were assessed *via *self-report questionnaire: The age dichotomy (< 35; > 35) was selected based on ages of men at greatest TC risk; race (white; other); marital status (divorced/single/unknown; married); education (< high school; at least some college; post- graduate); relation to a TC case (first-degree relative; second-degree relative); and prior history of testicular abnormality (cryptorchidism, orchitis, epididymitis, or injury to testicle; no abnormalities).

#### Knowledge of TC

We measured knowledge regarding TC with the Testicular Cancer Knowledge Scale (TCKS), a 10-item scale (range of scores = 0–10 determined by the sum of questions answered correctly) based on an instrument developed to assess TC-related knowledge among young adults (Cronbach's alpha = .70) [20], including items regarding TC etiology, the timing and method of TSE, and the sequelae of TC. Each item had 3 possible answers: Agree, Disagree, or Don't Know.

#### Health Belief Model Variables

We evaluated critical HBM concepts: perceived TC susceptibility and severity, as well as perceived benefits and barriers to TSE. In keeping with more recent HBM applications [32,33], we included the Cue to Action construct. We also included the health locus of control concept to assess individual belief that health is controlled by internal or external factors, such as performing self-examinations for cancer.

#### Perceived Susceptibility to Testicular Cancer Scale

Perceived susceptibility was assessed using previously-validated items associated with developing testicular [19] and breast cancer [34]. Perception of TC susceptibility was measured by summing responses to 3 five-point Likert scale items (1 = strongly agree to 5 = strongly disagree) (Cronbach's alpha = 0.76). An example of an item from this scale: *"It is likely that I will get testicular cancer."*

#### Perceived Severity of Testicular Cancer Scale

Perceived severity was assessed using previously-validated items associated with developing testicular cancer [19]. However, due to the low Cronbach's alpha for this scale in our sample [40], we included each item as a separate variable in the bivariate analyses. Thus, perception of severity was measured using individual values of three items with a five-point Likert scale (1 = strongly agree to 5 = strongly disagree). An example of an item from this scale: *"If I got testicular cancer, its impact on my life would be severe."*

#### Perceived Benefits of Testicular Self-examination

Perceived benefits were assessed using responses to 6 five-point Likert scale items (1 = strongly agree to 5 = strongly disagree) derived from previous health beliefs research regarding testicular [19] and breast cancer [34] risk (Cronbach's alpha = 0.77). An example of an item from this scale: *"If I examined my own testicles regularly, I might find a lump sooner that if I just went to the doctor for a check up."*

#### Perceived Barriers

Perceived barriers were assessed using responses to 7 five-point Likert scale items (1 = strongly agree to 5 = strongly disagree) derived from previous health beliefs research regarding testicular [19] and breast cancer risk [34] (Cronbach alpha = 0.61).). An example of an item from this scale: *"I am too busy to do TSE."*

#### Cue to Action for TSE

Previous cancer prevention behavior studies identified physician recommendation as a key factor predicting uptake of cancer self-examination behaviors. We adapted a single 2000 National Health Interview Survey item to assess whether a physician had ever recommended performing TSE (yes, no).

#### Locus of Control

The Multidimensional Health Locus of Control (MHLC) Scale consists of 3 six-point Likert scales (total: 18 items) (1 = strongly agree to 6 = strongly disagree). Internal Health Locus of Control (IHLC) quantifies the belief that internal factors are responsible for health/illness (Cronbach's alpha = .78); Powerful Others Health Locus of Control (PHLC) quantifies the belief that one's health is determined by powerful others (Cronbach's alpha = .57); and Chance Health Locus of Control (CHLC) quantifies the belief that health illness is a matter of fate, luck or chance (Cronbach's alpha = .62). [35]

#### Psychological Variables

We measured cancer worry and cancer distress (intrusive and avoidant thoughts).

#### Cancer Worry

We modified the Lerman Breast Cancer Worry Scale to assess cancer worry among men at risk of TC [36]. The scale assesses frequency of concerns about developing testicular cancer and the impact of cancer worry on mood and daily functioning on a 4-point scale (1 = not at all or rarely, to 4 = a lot) (Cronbach's alpha = 0.82) [36].

#### Cancer Distress via the Impact of Events Scale (IES)

The IES [37] measures the subjective impact of a specific event on an individual by assessing two major responses to stressful events: intrusion and avoidance. Intrusion is characterized by repetitive thoughts, mental images, disturbing dreams, and repetitive behavior, and is scored from 0 – 35 (Cronbach's alpha = .90). Avoidance is associated with denial of consequences from an event, blunted feelings, and emotional numbness related to an event [37], and is scored from 0 – 40 (Cronbach's alpha = .90). Respondents used a 4-point response scale (0 = 'not at all', 1 = 'rarely', 3 = 'sometimes', 5 = 'often') from a set of 15 TC-related statements to report the frequency with respect with which each occurred during the prior 7 days. It is a reliable and valid instrument for cancer-related distress among men and women either affected by or at risk of various cancers [38].

#### Practice of TSE

This was the primary analysis outcome. In the questionnaire, TSE was described as follows: "*Testicular Self-examination (TSE) is when a man checks himself for any abnormalities in his testicles*." Participants were then asked to provide a numerical response to the question "*Have many times during the past year have you performed TSE?*". We then classified men into two groups: **those who regularly performed TSE **(≥ 6 times in the past year) and **those who did not regularly perform TSE **(0–5 times in the past year). Previously- published research on TSE practices has used various standards to classify an individual as a regular performer of TSE, ranging from simply asking an individual whether they regularly perform TSE (yes, no) without defining the term "regular" [39], to categories based on a numeric response to a question about the frequency of TSE in a specified time frame [19,26]. However, the cut points for categorizing an individual as a regular TSE performer differ among studies. For example, one study considered an individual who reported performing TSE 1 to 10 times a year as a regular performer [26], while another considered only individuals who reported practicing TSE 12 times in the previous year as a regular performer [19]. There is current a lack of consensus among leading health and professional organizations on the recommended interval for TSE screening, and a lack of consistency in research studies regarding who is considered to be a "regular performer." Thus, we opted for a moderate approach whereby individuals who reported TSE > 6 times in the previous year were classified as regular performers, and those who reported TSE < 6 times as non-regular performers of TSE.

### Data Analysis

The main outcome of interest was Practice of TSE, a dichotomous variable consisting of those who reported they had regularly performed TSE (> 6 times in the previous year) compared to those who did not (< 6 times in the previous year). Descriptive statistics and reliability analyses were performed using the SPSS v. 15.0 statistical software package, and multivariate logistic regression employed SAS version 9.1.3. All analyses used two-tailed tests of significance. First, univariate analysis was used to compare TSE regular performers *versus *non-regular performers based on sociodemographic, medical, knowledge, HBM, and psychological factors. Independent variables that were significant at p < 0.05 (T-test, Chi square test) were then simultaneously entered into a multiple logistic regression model. Although the individual was the unit of analysis in this study, the familial nature of the cohort raised concerns regarding the assumption of observation independence. Therefore, all analyses that involved statistical testing (e.g. chi-square or t tests, logistic models) were conducted with a program that accounted for possible clustering within families (SAS Proc Surveyfreq, Proc Surveymeans, Proc Surveylogistic; SAS Institute, 2003). These procedures use the Taylor linearization method to estimate sampling errors of estimators based on potential clustering of responses between family members. Respondents with missing values for relevant variables were excluded from analyses.

## Results

### Respondent Characteristics

The majority of respondents were white, ≥ age 35, well-educated, and married (Table 1). Seventy-four men were first-degree, and 22 men were second-degree relatives of a case. Thirteen men reported having at least one testicular abnormality (e.g. cryptorchidism, orchitis, epididymitis, injury to testicle).

**Table 1:** Demographic and Medical Characteristics of Unaffected Men from Multiple-Case Testicular Cancer Families^a^

Factor	Total (n = 96)^b, c^	Regularly Performed TSE (n = 46)^c, d^	Did Not Regularly Performed TSE (n = 50)^c, e^	P-value*
Age (%; n)				
< 35	28.2 (28)	45.7 (21)	14.0 (7)	< 0.001
> 35	68.7 (68)	54.3 (25)	86.0 (43)	

Marital Status (%; n)				
Divorced/Single/Widowed/Unknown	33.3 (33)	39.1 (18)	30.0 (15)	0.38
Married	66.6 (63)	60.1 (28)	70.0 (35)	

Race (%; n)				
White	92.0 (91)	93.5 (43)	96.0 (48)	0.14
Other	4.0 (4)	6.5 (3)	2.0 (1)	

Education (%; n)				
<=high school	16.2 (16)	17.4 (8)	16.0 (8)	0.97
Some college/college graduate	47.5 (47)	50.0 (23)	48.0 (24)	
Post graduate	32.3 (32)	32.6 (15)	34.0 (17)	

Relation to Case (%; n)				
First-Degree Relative	74.7 (74)	91.3 (42)	64.0 (32)	< 0.01
Second-Degree Relative	22.2 (22)	8.7 (4)	36.0 (18)	

Prior history of TC abnormality (cryptorchidism, orchitis, epididymitis, or injury to testicle) (%; n)				
Yes	13.1 (13)	15.2 (7)	12.0 (6)	0.68
No	82.8 (82)	84.8 (39)	86.0 (43)	

Physician Rec. (Cue to Action) (%; n)				
Yes	40.4 (40)	69.6 (32)	16.0 (8)	< 0.001
No	56.6 (56)	30.4 (14)	84.0 (42)	

^a^For dichotomous variables, *X*^2 ^test of heterogeneity was used to compare groups using the Taylor Linearization method to adjust for possible familiar clustering; ^b^Three subjects did not respond to the question about TSE and thus were not included in the bivariate analysis; ^c^Percentages may not total to 100 due to missing data; ^d^Regularly performed TSE = those reporting ≥ 6 TSEs in the previous year; ^e^Did not regularly perform TSE = those reporting < 6 TSEs in the previous year

### Performance of TSE

Of the 99 participants who met inclusion criteria for this sub-study, forty-six percent reported performing TSE six or more times in the previous year, while 51% reported performing TSE less than 6 times; the remaining 3% did not respond to this question.

### Individual Factors Associated with Regular Performance of TSE

Demographic and medical factors positively-correlated (p < 0.05) with regular performance of TSE were older age (> 35), being a first-degree relative of a family member with testicular cancer, and having a physician recommend TSE (Table 1). Additionally, having greater TC knowledge, perceiving greater benefits and fewer barriers related to performing TSE, having higher levels of cancer worry, and intrusive thoughts related to testicular cancer were associated with regular performance of TSE (Table 2).

**Table 2:** Knowledge, Health Belief and Psychosocial Characteristics of Unaffected Men from Multiple-Case Testicular Cancer Families^a^

Factor	Total (n = 96)^b, c ^(X+SE)	Regularly Performed TSE^b, c ^(n = 46) (X+SE)	Did Not Regularly Perform TSE^b, d ^(n = 50) (X+SE)	P-value*
Testicular Cancer Knowledge	4.2 (0.3)	5.0 (0.4)	3.5 (0.4)	0.02

Perceived Benefits	24.9 (0.4)	26.4 (0.6)	23.5 (0.5)	< 0.01

Perceived Barriers	19.0 (0.5)	17.2 (0.5)	20.5 (0.5)	< 0.001

Perceived Severity (impact of TC on life)	3.3 (0.1)	3.4 (0.2)	3.2 (0.2)	0.47

Perceived Severity (pain and suffering from TC)	3.3 (0.1)	3.3 (0.2)	3.4 (0.1)	0.52

Perceived Severity (chance of survival from TC)	2.6 (0.2)	2.5 (0.3)	2.7 (0.3)	0.50

Perceived Susceptibility	8.5 (0.2)	8.9 (0.3)	8.1 (0.3)	0.06

Internal Health Locus of Control	25.2 (0.5)	26.0 (0.7)	24.6 (0.6)	0.13

Powerful Others Health Locus of Control	18.9 (0.3)	18.8 (0.5)	19.0 (0.6)	0.84

Chance Health Locus of Control	17.8 (0.4)	17.1 (0.7)	18.4 (0.6)	0.19

Cancer Worry	5.7 (0.3)	6.5 (0.4)	5.0 (0.2)	2

Intrusion	2.1 (0.5)	3.2 (0.9)	1.0 (0.4)	0.03

Avoidance	3.4 (0.7)	4.6 (1.3)	2.2 (0.6)	0.09

^a^For continuous variables, independent samples t-test used to compare groups using the Taylor Linearization method to adjust for possible familiar clustering; ^b^Three subjects did not respond to the question about practice of TSE and thus were not included in the bivariate analysis; ^c^Regularly performed TSE = those reporting ≥ 6 TSEs in the previous year; ^d^Did not regularly perform TSE = those reporting < 6 TSEs in the previous year

Factors not significantly associated with regular performance of TSE included marital status, education, having a prior testicular abnormality, perceived TC susceptibility, Health Locus of Control (internal, powerful others, or chance), and having avoidant thoughts.

Multiple logistic regression analyses incorporating the variables above that were significant in the univariate analyses were conducted both with (Proc Survey Logistic Regression) and without adjusting for familial clustering, with similar results for both methods. The results adjusting for familial clustering are presented in Table 3. The variables that remained significantly associated (p < .05) with regular performance of TSE were physician recommendation for performing TSE (OR = 6.6, 95% CI 2.0, 21.1) and having higher levels of cancer worry about TC (OR = 1.6, 95% CI 1.1, 2.3).

**Table 3:** Logistic Regression Model of Predictors of Testicular Self-examination among Unaffected Men from Multiple-Case Testicular Cancer Families (n = 91)

Factor	Odds Ratio (95% Confidence Interval)
Age > 35	0.4 (0.1, 1.3)

FDR of Case	2.2 (0.7, 8.0)

Physician Recommendation for TC	6.6 (2.0, 21.1)*

Testicular Cancer Knowledge	1.2 (0.84, 1.6)

Perceived Benefits	1.1 (0.9, 1.4)

Perceived Barriers	0.8 (0.6, 1.0)

Cancer Worry	1.6 (1.1, 2.3)*

Intrusion	1.0 (0.9, 1.1)

*significant at p < .05

## Discussion

Forty six percent of unaffected, at-risk men from multiple-case TC families in our sample reported regular performance of TSE. The two factors significantly associated with regular performance of TSE were physician recommendation and higher levels of TC cancer worry.

In a 2005 review of TSE practices that included 6 studies of U.S. men ≥ age 18 [39], the percent of men practicing TSE on a monthly basis ranged from 6 to 36% Our TSE rates are not directly comparable to those data, which defined regular performance of TSE as once a month, since we used a broader inclusion for our regular performer category of 6 times a year or more. This may explain the higher proportion of men in our sample that were defined as regular performers of TSE (46%).

With respect to the HBM variables assessed in the current study, only Cues to Action (i.e. physician recommendation for TSE), remained a statistically significant predictor of TSE behavior in multivariate analysis. The importance of physician recommendation on TSE practice was striking, with men who reported a physician recommendation for TSE having at least six times higher odds of performing regular TSE compared with those who reported no such recommendation. This provides yet another example [33,40,41] of how potent an influence the physician can be with regard to cancer prevention behavior in patients, and underscores the importance of physicians including such recommendations in the course of ongoing health care discussions. Due to the lack of a strong evidence base to support the belief that TSE is associated with improved TC-related survival among those who practice it, and the remarkable therapeutic successes in managing TC patients even with far-advanced disease, it is unlikely that there ever will be a clinical trial testing this question. In this era of evidence-based medical practice, it is difficult for health care providers to insist that at-risk men adopt TSE practice, although common sense and logic suggest that a TC detected by TSE rather than symptoms should be more amenable to treatment regimens that do not include chemotherapy, or permit less intense or shorter chemotherapy regimens. This rationale is not unlike recommendations for both BSE, CA-125 testing, and transvaginal ultrasound recommendations for women at increased risk for hereditary breast and ovarian cancer [42]. Given that TC is a disease which affects young men during the most productive period of their lives, there is potential for real economic and psychosocial benefit in attempting to minimize treatment-associated costs and morbidity.

Two previous studies of the HBM and TSE showed that perceived benefits and barriers were associated with TSE, as did our data [19,29]. In our study, perceived cancer susceptibility was not associated with TSE. Although one previous study examining the relationship between TSE and perceived susceptibility found a significant association, there were only 12 men in the entire study who were considered to be "practicers" of TSE (i.e. reported practicing TSE > 4 times a year) [19,29]. A larger study of TSE behaviors using multivariate analyses failed to replicate this finding [29]. Perceived severity of TGCT was not a significant predictor of PSA testing. This finding is similar to two previous studies of TSE that used the HBM as a theoretical framework [19,29]. Previous cancer screening research for a variety of sites has also found perceived susceptibility of little predictive value with regard to screening behaviors, possibly due to the almost universally held belief that cancer is a severe disease, regardless of type [43-48]. In our study, none of the HBM factors significant in bivariate analyses remained so when they were considered in a multivariate model which included physician recommendation. This result is consistent with many previous studies of cancer screening behaviors.

To our knowledge only one other study has examined HBM with respect to TSE using multivariate analysis. Overall, it appears that the HBM does not adequately explain TSE behavior. Similar to our study, HBM variables accounted for only a small proportion of the variance in TSE behavior (21%) [29]. As discussed below, cancer-related worry was the main psychosocial predictor of TSE behavior in our study and may lend some support to the commonly-raised concern regarding the HBM and other cognitive models of health behavior, namely that they do not include affective variables as behavior predictors [30].

Cancer-related worry was the other major factor associated with TSE practice patterns in our study. From a theoretical perspective, several relationships between cancer worry and cancer prevention behaviors have been proposed. Some models posit that worry may serve as a facilitator to cancer screening behavior, others postulate worry acts a barrier, and some propose a curvilinear relationship between cancer worry and preventive behaviors, whereby worry is a facilitator to a certain level after which it becomes a barrier [49]. The HBM, which was the theoretical basis of the present study, has been used to support the role of worry as both a barrier and facilitator for cancer screening behavior [49]. In our sample, it appears that cancer worry motivates individuals to practice TSE, since those with higher levels of cancer worry were also more likely to perform TSE. However, the directionality of this relationship can only be established in the context of a prospective study; our data are cross-sectional. Studies in other high-risk populations provide some evidence that cancer worry may in fact ***precede ***screening behavior. In a review of the relationship between cancer worry and mammography screening among high-risk women, four of five *prospective *studies indicated that higher levels of cancer worry were associated with greater rates mammography screening and breast self-examination [49]. However, no similar prospective studies of high-risk men are available. One cross-sectional study of prostate cancer screening among a sample of men at increased risk of hereditary prostate cancer found a negative relationship between cancer worry and screening behavior [50].

It is noteworthy that we observed no statistically significant differences in TSE practices between men with and without a prior history of a testicular abnormality. A personal history of cryptorchidism can increase the risk of TC up to 11-fold [51]. Of the 13 men reporting a testicular abnormality in our series, 29% (n = 4) reported a history of cryptorchidism (data not shown). In a study by Blesch and colleagues, none of the 5 men with a history of cryptorchidism, reported regularly practicing TSE [19]. While this finding may reflect the small number of men with this particular risk factor and the associated lack of statistical power to detect a difference in that study, it is possible that men may be unaware that testicular maldescent is among the most strongly-established TC risk factors. However, no study to date has focused specifically on the levels of awareness/knowledge of cryptorchidism as a risk factor for TC or as a multivariate predictor of TSE behavior.

In multivariate analyses, men age = 35 and those who were first-degree relatives of a TC case were no more likely to perform TSE than were older men or those whose affected family member was a more distant relative. In the 2005 review mentioned above [27], only 12–25% of participants in U.S. studies were aware of the age group most affected by TC. In the study by Blesch and colleagues [19], men with a family history of TC were significantly more likely to perceive greater benefits to TSE. However, the study did not assess the impact on actual TSE behavior. Taken together, the non-differential rates of TSE between men in younger and older age groups, men who are first *versus *second-degree relatives of a TC case, and men with a prior testicular abnormality compared with those without, suggest a lack of awareness on the part of unaffected family members regarding the additional risk conferred by having these characteristics. It is also possible that men recognize the implications of these characteristics, but they are not sufficiently concerned by this information to motivate screening behavior.

To our knowledge, this is the first study to examine TSE behaviors among unaffected men from multiple-case testicular cancer families. While the results provide important information about current rates and correlates of TSE in this high-risk group, they should be interpreted with caution due to certain limitations of our study. A goal of our study was to evaluate the utility of HBM in understanding TSE behavior. Ideally, use of an analytic technique such as structural equation modeling would allow us to definitively test the utility of HBM in predicting TSE behavior. However, our study was designed as a pilot study of the utility of HBM and it did not have a sample size sufficient to reach definitive conclusions. It is possible that the HBM did not adequately capture those factors that were important in TSE behavior. For example, one of the major criticism of HBM and other cognitive models of health behavior is the lack of inclusion of affective variables (e.g. distress) [30]. To address this issue, we included scales for both cancer worry and cancer distress. Additionally, we included demographic and SES variables that may also impact screening behavior. Finally, in a study that compared the HBM to the Theory of Planned Behavior, the quality of the models was very similar. The TPB explained 22%of the variance in behavior *versus *21% by the HBM, suggesting that applying another theoretical framework may have yielded similar results [29]. Thus, based on our review of the literature related to theories of health behavior and predictors of screening, we feel that we adequately expanded the HBM to include other factors associated with TSE behavior. In addition, this was a cross-sectional study, making it difficult to establish that the beliefs and psychological factors we identified preceded the behavior, as hypothesized in the HBM. Additionally, men volunteering to participate in a study of familial TC may be more aware of and concerned about TC. Thus, the rates of TSE may be higher in this study population than men with a similar history not participating in such a study. Similarly, these men may be more knowledgeable about TC and have differing beliefs and psychological responses to TC.

As molecular genetic diagnosis improves, family history of TC will become an increasingly important risk factor on which to base decisions for primary, secondary, and tertiary prevention of TC. The present study showed that among men with a family history of TC, those who had higher levels of cancer worry were more likely to practice TSE regularly. However, findings also revealed that men at increased risk of TC due to a positive family history may be either less knowledgeable about and/or less motivated to perform TSE even if they are younger, have a prior testicular abnormality, or are a FDR of a man with TC. Thus, these are areas in which men may benefit from additional education and information upon which to base decisions about TSE practices. Physician recommendation emerged as the most important predictor of regular performance of TSE. Despite the absence of proof regarding the efficacy of TSE, it nonetheless remains prudent to encourage health care providers to recommend monthly TSE for high-risk patients, and to instruct their patients in performing this examination. Given the superficial/accessible location of the testes, and the existence of non-invasive imaging (testicular ultrasound) and tumor marker assays, the price one pays for a false-positive screening examination is likely to be substantially less than would be the case for ovarian cancer screening, for example. Finally, providing at-risk men information regarding accepted TC risk factors, as a means of deepening their understanding of their own risk, might help to correct some of the information deficiencies we identified. Despite the limitations in both our and other's studies, we have adopted the policy of recommending monthly TSE as a screening modality for unaffected bloodline males from multiple-case TC families, while clearly informing our patients that this represents our best clinical judgment rather than an evidence-based recommendation.

## Competing interests

The authors declare that they have no competing interests.

## Authors' contributions

STV designed original sub study protocol and drafted manuscript

RPM conducted all statistical analyses and participated in manuscript preparation

JL participated in design of original sub study protocol and manuscript preparation

JAP participated in design of original sub study protocol and manuscript preparation

MHG conceived and designed original main research protocol and participated in manuscript preparation

LK oversaw data collection and participated in manuscript preparation

All authors read and approved the final manuscript.
